# Supplementary material for: An Update on the Efficacy of Single and Serial Intravenous Ketamine Infusions and Esketamine for Bipolar Depression: A Systematic Review and Meta-Analysis
Source: Brain Sci. 2023 Dec 2;13(12):1672. doi: 10.3390/brainsci13121672 (PMC10741553; doi:10.3390/brainsci13121672)
Supplement: Supplementary file 1 [file brainsci-13-01672-s001.zip › brainsci-2722059-supplementary.pdf]

APPENDIX A

Tables S1. Search Strategy.

Ovid MEDLINE(R) Epub Ahead of Print, In-Process & Other Non-Indexed Citations, Ovid MEDLINE(R) Daily and Ovid MEDLINE(R) <1946 to Present> Search history sorted by search number ascending

#	Searches	Results
1	Bipolar Disorder/	44640
2	Depression/	146981
3	depressive disorder/ or depressive disorder, major/ or depressive disorder, treatment-resistant/	112257
4	((manic adj1 depress*) or antidepress* or "anti-depress*" or "Bipolar depression" or bipolar* or depression* or "treatment resistant bipolar depression" or "treatment resistant depression").mp.	606217
5	or/1-4	626805
6	KETAMINE/	14609
7	(arketamine or esketamine or ketamine or "r-ketamine" or "s-ketamine").mp.	23536
8	6 or 7	23536
9	5 and 8	4370
10	limit 9 to (adaptive clinical trial or clinical study or clinical trial, all or clinical trial, phase i or clinical trial, phase ii or clinical trial, phase iii or clinical trial, phase iv or clinical trial or comparative study or controlled clinical trial or equivalence trial or evaluation studies or meta analysis or multicenter study or observational study or pragmatic clinical trial or randomized controlled trial or systematic reviews) [Limit not valid in Ovid MEDLINE(R),Ovid MEDLINE(R) Daily Update,Ovid MEDLINE(R) PubMed not MEDLINE,Ovid MEDLINE(R) In-Process,Ovid MEDLINE(R) Publisher; records were retained]	838
11	9 and ("case control*" or cohort* or "cross section*" or prospective* or retrospective* or trial*).mp.	1289
12	10 or 11	1493

EBM Reviews - Cochrane Central Register of Controlled Trials January 2023

#	Searches	Results
1	Bipolar Disorder/	3228
2	Depression/	15696
3	depressive disorder/ or depressive disorder, major/ or depressive disorder, treatment-resistant/	13966
4	((manic adj1 depress*) or antidepress* or "anti-depress*" or "Bipolar depression" or bipolar* or depression* or "treatment resistant bipolar depression" or "treatment resistant depression").mp.	102653
5	or/1-4	103427
6	KETAMINE/	2667
7	(arketamine or esketamine or ketamine or "r-ketamine" or "s-ketamine").mp.	6674
8	6 or 7	6674

9	5 and 8	1530
10	limit 9 to (adaptive clinical trial or clinical study or clinical trial, all or clinical trial, phase i or clinical trial, phase ii or clinical trial, phase iii or clinical trial, phase iv or clinical trial or comparative study or controlled clinical trial or equivalence trial or evaluation studies or meta analysis or multicenter study or observational study or pragmatic clinical trial or randomized controlled trial or systematic reviews) [Limit not valid in CCTR; records were retained]	1530
11	9 and ("case control*" or cohort* or "cross section*" or prospective* or retrospective* or trial*).mp.	1071
12	10 or 11	1530

Embase 1988 to 2023 Week 05

#	**Searches**	**Results**
1	Bipolar Disorder/ or exp bipolar depression/	67447
2	Depression/	428490
3	depressive disorder/ or depressive disorder, major/ or depressive disorder, treatment-resistant/	203528
4	((manic adj1 depress*) or antidepress* or "anti-depress*" or "Bipolar depression" or bipolar* or depression* or "treatment resistant bipolar depression" or "treatment resistant depression").mp.	917287
5	or/1-4	917643
6	KETAMINE/	48682
7	(arketamine or esketamine or ketamine or "r-ketamine" or "s-ketamine").mp.	52972
8	6 or 7	52972
9	5 and 8	10341
10	limit 9 to (adaptive clinical trial or clinical study or clinical trial, all or clinical trial, phase i or clinical trial, phase ii or clinical trial, phase iii or clinical trial, phase iv or clinical trial or comparative study or controlled clinical trial or equivalence trial or evaluation studies or meta analysis or multicenter study or observational study or pragmatic clinical trial or randomized controlled trial or systematic reviews) [Limit not valid in Embase; records were retained]	1734
11	9 and ("case control*" or cohort* or "cross section*" or prospective* or retrospective* or trial*).mp.	3643
12	10 or 11	3679
13	12 not case report	

APA PsycInfo 1806 to January Week 5 2023

#	**Searches**	**Results**
1	Bipolar Disorder/ or exp bipolar depression/	29808
2	exp Treatment Resistant Depression/	2883
3	exp Major Depression/	153509
4	((manic adj1 depress*) or antidepress* or "anti-depress*" or "Bipolar depression" or bipolar* or depression* or "treatment resistant bipolar depression" or "treatment resistant depression").mp.	423613
5	or/1-4	423843

6	exp Ketamine/	2753
7	(arketamine or esketamine or ketamine or "r-ketamine" or "s-ketamine").mp.	4592
8	6 or 7	4592
9	5 and 8	2105
10	limit 9 to all journals	1993
11	limit 10 to human	1189

Scopus

((TITLE-ABS-KEY ((arketamine OR esketamine OR ketamine OR "r-ketamine" OR "s-ketamine") W/10 ((manic AND adj1 AND depress*) OR antidepress* OR "anti-depress*" OR "Bipolar depression" OR bipolar* OR depression* OR "treatment resistant bipolar depression" OR "treatment resistant depression")) AND TITLE-ABS-KEY (trial* OR random* OR cohort* OR controlled OR compar* OR prospective* OR retrospective* OR study OR studies OR systematic OR meta-analysis) AND NOT TITLE-ABS-KEY (cats OR mice OR rats OR rabbit* OR child* OR pediatr* OR paediatr*)) **1546**

Table S2: Cochrane risk-of-bias tool for randomized trials

Criteria	***Diazgranados et al, 2010***	***Zarate et al, 2012***	***Grunebaum et al, 2016***
Random sequence generation (selection bias)	Low risk.	Low risk.	Low risk.
Allocation concealment (selection bias)	Low risk.	Low risk.	Low risk.
Blinding of participants and personnel	Unclear risk.	Unclear risk.	Unclear risk.
Blinding of outcome assessment (detection bias)	Unclear risk.	Unclear risk.	Unclear risk.
Incomplete outcome data addressed (attrition bias)	Low risk.	Low risk.	Low risk.
Selective reporting (reporting bias)	Unclear risk.	Unclear risk.	Unclear risk.
Other bias	Unclear risk.	Unclear risk.	Unclear risk.

Table S3. Quality assessment of the open-label studies- Methodological Index For Non-Randomized Studies (MINORS)

Methodological items for non-randomized studies		Score†							
		Kantrowitz,et al., 2015	Rybakowski, et al., 2017	Zhou et al., 2020	Zheng et al., 2020	Wilkowska et al., 2021	Fancy et al., 2023	Delfino et al., 2021	Martinotti et al., 2023
1	**A clearly stated aim**: the question addressed should be precise and relevant in the light of available literature	2	2	2	2	2	2	2	2
2	**Inclusion of consecutive patients**: all patients potentially fit for inclusion (satisfying the criteria for inclusion) have been included in the study during the study period (no exclusion or details about the reasons for exclusion)	2	2	0	0	0	0	0	0
3	**Prospective collection of data**: data were collected according to a protocol established before the beginning of the study	2	2	2	2	2	2	2	2
4	**Endpoints appropriate to the aim of the study**: unambiguous explanation of the criteria used to evaluate the main outcome which should be in accordance with the question addressed by the study. Also, the endpoints	2	2	2	2	2	2	2	2

	should be assessed on an intention-to-treat basis.								
5	**Unbiased assessment of the study endpoint**: blind evaluation of objective endpoints and double-blind evaluation of subjective endpoints. Otherwise the reasons for not blinding should be stated	0	0	0	0	0	0	2	2
6	**Follow-up period appropriate to the aim of the study**: the follow-up should be sufficiently long to allow the assessment of the main endpoint and possible adverse events	2	2	2	2	2	2	2	2
7	**Loss to follow up less than 5%**: all patients should be included in the follow up. Otherwise, the proportion lost to follow up should not exceed the proportion experiencing the major endpoint	2	2	2	2	2	2	2	2
8	**Prospective calculation of the study size**: information of the size of detectable difference of interest with a calculation of 95% confidence interval, according to the expected incidence of	0	0	0	0	0	0	0	2

	the outcome event, and information about the level for statistical significance and estimates of power when comparing the outcomes								

†The items are scored 0 (not reported), 1 (reported but inadequate) or 2 (reported and adequate). The global ideal score being 16 for non-comparative studies.

Figure S1. Risk of bias for RCTs Included in systematic review.

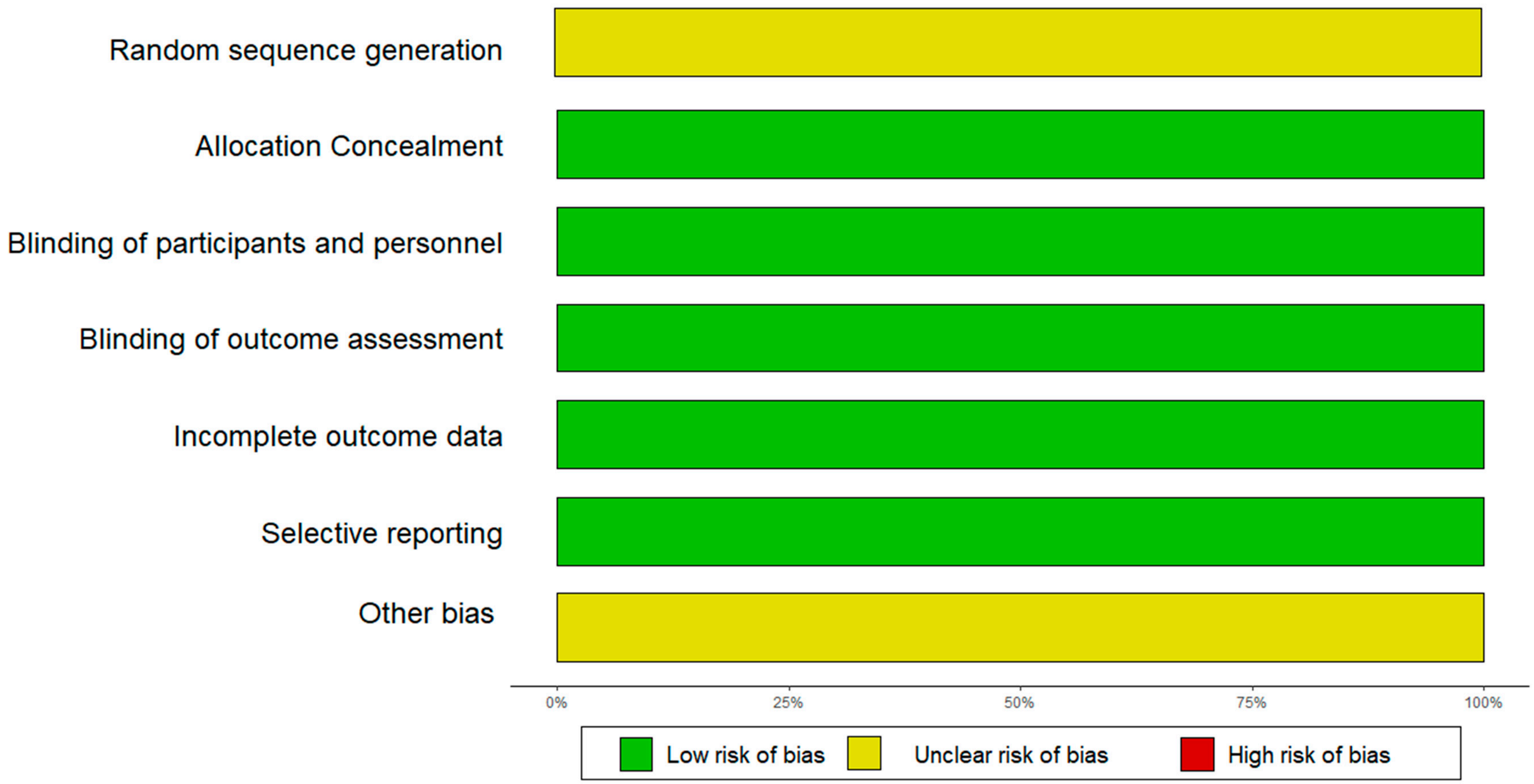

Table S4. Inclusion/Exclusion Criteria and Adverse Events (AEs)

References	*Inclusion Criteria*	*Exclusion Criteria*	*Adverse Events (AEs)*
**Diazgranados N, et al.**<br>**2010**	-Women (18-65 years old) with BD-I or BD-II with a depressive episode MADRS >20 and failed at least one antidepressant trial.	-Comorbid substance abuse<br>-Previous treatment with ketamine<br>-Treatment with other mood stabilizers beside lithium or valproate 2 weeks before randomization.<br>-Pregnancy or lactation	-No serious AEs.<br>-10% in both groups during infusion reported feeling drowsy, blurred vision, headache, nausea, anxious, lethargic or cognitively impaired.<br>-In ketamine group 10% reported dry mouth, palpitations, increased blood pressure with bizarre feelings and dissociation.
**Zarate CA Jr, et al.**<br>**2012**	-Adult patients (18 to 65 years old) diagnosed with BD-I or BD-II without psychotic features with a MDE ≥ 4 weeks duration.<br>-MADRS ≥ 20 before randomization.<br>- Failure of at least one antidepressant trial or a failed a prospective open trial of a mood stabilizer.	Comorbid substance abuse or dependence for at least 3 months before enrollment,<br>-Serious unstable medical condition<br>-Previous treatment with ketamine<br>- Treatment with other mood stabilizers beside lithium or valproate 2 weeks before randomization.<br>- Pregnancy or lactation	-No serious AEs.<br>-10% during infusion receiving ketamine or placebo reported: feeling drowsy, blurred vision, headache, nausea, anxious, lethargic or cognitively impaired.<br>-No adverse event was significantly different from placebo at 80 minutes or thereafter.<br>-10% in both groups reported headaches, drowsiness or sedation, early morning awakening, and difficulty falling asleep.<br>-Ketamine group reported: dry mouth, dizziness or faintness, difficulty falling asleep, and flatulence were reported for ketamine only<br>-Placebo group reported: irritability and muscle, bone, or joint pain.<br>-No significant changes occurred in electrocardiogram, respiratory, or laboratory values during the study.

**Kantrowitz JT, et al., 2015**	-Adult patients (18 to 60 years old) diagnosed with BD-I or BD-II without psychotic features with a MDE ≥ 4 weeks duration.<br>-MADRS ≥ 20 before randomization.<br>-Insufficient therapeutic response during the current episode.	-Alcohol abuse or dependence<br>-Current or chronic psychosis<br>-Drug induced psychosis<br>-Drug abuse/dependence in the last six months<br>-Current suicide or violence risk<br>-Pregnancy and lactation	No serious adverse events.
**Grunebaum, et al. 2017**	-Patients 18−65 years old with DSM-IV BD criteria experiencing a major depressive episode with score ≥16 in the HDRS-17.<br>-Voluntary admission to inpatient research unit at the New York State Psychiatric Institute for the infusion.	-Unstable medical condition<br>-Pregnancy or lactation<br>-Current psychosis<br>-Ketamine abuse/dependence<br>-Drug/alcohol dependence within 6 months<br>- Suicidality due to binge substance use/withdrawal<br>-Ineffective trial or adverse reaction to ketamine/midazolam<br>-Daily opiate use or equivalent for 3 days pre-infusion<br>-Mini Mental State Exam score < 25<br>-Lack of capacity to consent<br>-Inadequate understanding of English.	-There were no serious cardio-respiratory adverse events.<br>-Transient increase in systolic blood pressure was seen in the ketamine group.<br>-No infusion-related dissociative or psychotomimetic serious adverse events.<br>-Increased suicide ideation in one subject during the evening after ketamine infusion, which remitted over the subsequent 24 hours in what appeared to be a delayed ketamine response.<br>-Increased suicidal ideation during follow-up months 2 to 6 in two subjects (four events; three for one subject) and one suicide attempt during follow up month 2.
**Rybakowski JK, et al. 2017**	-Adults with BD (22–81 years old) receiving one or more mood stabilizing medications for at least one year.<br>-Lack of treatment response after at least two adequate courses of antidepressant and/or mood-stabilizing treatment within the previous three months<br>- HDRS-17 ≥18<br>-All antidepressants were discontinued for at least 7 days prior to infusion	-Any other psychiatric comorbidity and substance misuse disorders, except for alcohol abuse/dependence<br>-Pregnancy or serious medical condition.	-No serious AEs reported.<br>-Transient increase in blood pressure occurred in one third of the patients.<br>-Psychiatric side effects, such as depersonalization and derealization, were experienced by one-third of patients only during the infusion.

**Fancy et al., 2023**	- Adult patients >18 years of age<br><br>-Failure of at least two adequate medication trials<br>- Not having unstable medical conditions (uncontrolled hypertension), pregnancy or psychiatric contraindications (active psychosis or current substance use disorder)<br><br>- Patients with a substance use disorder were included if they were able to abstain from the given substance for 3 months prior to treatment	- Patients with a history of psychosis or current substance use disorders were excluded from receiving treatment	- Tolerability to ketamine was assessed through reported treatment-emergent adverse events (e.g., nausea, dizziness, depersonalization, etc.).<br><br>-Most frequently reported adverse events included: drowsiness (29.3%), dizziness (28.2%), blurred vision (26.8%), and confusion (26.8%).
**Zheng et al., 2020**	-Patients 18−65 years old with DSM-5 (SCID-5) BD/MDD criteria experiencing MDE without psychosis.<br><br>-MDE moderate to severe by score ≥17 in the HDRS-17 and SSI part I ≥ 2<br><br>-Ability to understand the study procedure and sign a written informed consent.	- Met DSM-5 criteria for schizophrenia, organic mental disorders, substance or alcohol use disorder<br><br>-Current psychotic symptoms<br>- a positive urine toxicology at screening<br><br>- Unstable medical illness<br><br>- Lifetime history of neurological diseases<br>- Pregnancy or lactation or planned pregnancy.<br><br>-Inability to follow the study procedures.	N/A
**Wilkowska et al., 2021**	-Patients 18−65 years old with DSM-IV BD criteria experiencing a major depressive episode with score ≥16 in the HDRS-17.<br><br>- Score ≥4 on the SSI	- Unstable medical or neurological illness<br><br>-Significant electrocardiogram (ECG) abnormality<br><br>- current psychosis<br><br>-history of ketamine abuse or dependence, other drug or alcohol dependence within 6 months<br><br>- suicidality due to binge substance use or withdrawal<br><br>-prior ineffective trial of or adverse reaction to ketamine or midazolam	-No serious adverse events associated with ketamine treatment<br><br>-Common side effects were dizziness, nausea, headache and insomnia

		- daily opiate use >20 mg oxycodone or equivalent during the 3 days pre-infusion<br>- score <25 on the Mini Mental State Exam18 (for subjects >60 years old)<br>-lack of capacity to consent and inadequate understanding of English.<br>-There was no exclusion for body mass index or weight.<br>- Pregnancy or lactation	
**Zhou et al., 2020**	-DSM-IV criteria for BD<br>-TRBD defined as failed attempts to achieve remission after 8 weeks each of 2 or more separate monotherapies or one monotherapy with one combination treatment.<br>- patients capable of providing informed consent.<br>- Failure of 2 trials of antidepressant treatment with a mood stabilizer or antipsychotic agent without alleviation of their depression symptoms<br>-HAMD-17 score of ≥24<br>-YMRS score ≤5<br>- Clinically stable on either a mood stabilizer and/or antipsychotic medication before entering the study	- Individuals with severe physical illnesses (such as atherosclerosis, diabetes, hypertension, infection, or epilepsy)<br>- Pregnant or planned pregnancy<br>- Individuals with any other major axis I disorders<br>-Individuals with a history of electroconvulsive therapy (ECT) in the past 6 months.<br>- Contraindications for MRI<br>-History of LOC > 5 minutes due to any cause<br>- Current suicidal ideation<br>-IQ < 80	N/A
**Martinotti et al., 2023**	-age over 18 years<br>-experiencing a depressive episode<br>- being treated with an SSRI or SNRI<br>-Tried and failed at least two adequate trials (in dosage achieved and duration) from 2 classes of antidepressants and two classes of mood stabilizers (including atypical antipsychotic agents).	-Comorbid organic pathologies such as untreated hypertension, previous cerebrovascular disorders.	-31.4 % experienced dissociation symptoms while 29% sedation.<br>-Additionally, anxiety, manic symptoms and dizziness reached approximately 3%.<br>-Overall, 57% of patients endorsed any side effect.

**Delfino et al.,**<br><br>**2021**	-major depressive episode in the context of major depressive disorder or bipolar depression according to DSM-IV criteria.<br><br>- score ≥ 25 points on MADRS<br><br>- failed to respond to ≥ 2 pharmacological treatments	N/A	N/A
**Abbreviations:** MADRS, Montgomery-Åsberg Depression Rating Scale; HDRS-17 or HAM-D:Hamilton Depression Rating Scale SSI, Scale for Suicidal Ideation MMSE, Mini Mental State Exam; LOC: loss of consciousness; YMRS: Young Mania Rating Scale; TRBD: Treatment resistant bipolar depression; AES: adverse events			
